# A Rapid and Efficient Luminescence-based Method for Assaying Phosphoglycosyltransferase Enzymes

**DOI:** 10.1038/srep33412

**Published:** 2016-09-14

**Authors:** Debasis Das, Marthe. T. C. Walvoort, Vinita Lukose, Barbara Imperiali

**Affiliations:** 1 Department of Biology and Department of Chemistry, Massachusetts Institute of Technology, Cambridge, MA 02139, USA

## Abstract

Phosphoglycosyltransferases (PGTs) are families of integral membrane proteins with intriguingly diverse architectures. These enzymes function to initiate many important biosynthetic pathways including those leading to peptidoglycan, N-linked glycoproteins and lipopolysaccharide O-antigen. In spite of tremendous efforts, characterization of these enzymes remains a challenge not only due to the inherent difficulties associated with the purification of integral membrane proteins but also due to the limited availability of convenient assays. Current PGT assays include radioactivity-based methods, which rely on liquid-liquid or solid-liquid extractions, multienzyme systems linked to lactate dehydrogenase and NAD^+^ generation, and HPLC-based approaches, all of which may suffer from low sensitivity and low throughput. Herein, we present the validation of a new luminescence-based assay (UMP-Glo) for measuring activities of PGT enzymes. This assay measures UMP, the by-product of PGT reactions, in a sensitive and quantitative manner by measuring the luminescence output in a discontinuous coupled assay system. The assay is rapid and robust in nature, and also compatible with microtiter plate formats. Activity and kinetic parameters of PglC, a PGT from *Campylobacter jejuni*, were quickly established using this assay. The efficacy of the assay was further corroborated using two different PGTs; PglC from *Helicobacter pullorum* and WecA from *Thermatoga maritima*.

Polyprenol-phosphate phosphoglycosyltransferases (PGTs) are integral membrane proteins that catalyze transfer of a C1-phospho-sugar from a nucleotide diphosphate sugar to polyprenol-phosphate, forming a polyprenol-diphospho-sugar product with concomitant release of a nucleotide monophosphate (NMP). These enzymes are often referred to as “priming” glycosyltransferases due to their essential functions in initiating biosynthesis of glycans and glycoconjugates^1^,^2^ including glycoproteins and glycolipids^3^. In bacteria, these glycoconjugates are commonly found at cell surfaces where they are important in mediating interactions with host cells and the environment. For example, in bacteria, WecA is a PGT that catalyzes the first step in the biosynthesis of lipopolysaccharide O-antigen^4^, and MraY catalyzes formation of the first membrane-associated intermediate in peptidoglycan biosynthesis^5^. In the eukaryotic dolichol pathway^6^, Alg7 is a PGT that initiates N-linked protein glycosylation by transferring phospho-*N*-acetylglucosamine to a dolichol-phosphate acceptor. Protein glycosylation through the dolichol pathway has critical and fundamental implications for the stability and functions of the modified proteins, and defects in this pathway, including Alg7, result in severe and often lethal developmental defects^7^.

To date, biochemical and bioinformatics studies have highlighted conserved motifs and residues crucial for catalytic activity in different PGTs^4^,^8^,^9^,^10^,^11^, and recently the X-ray structure of MraY has been reported, providing the first structural snapshot of a PGT^12^. However, despite their centrality in glycoconjugate biosynthesis, the characterization of PGTs remains a challenge, due to difficulties associated with overexpressing and purifying integral membrane proteins, and because of limited access to robust assays that can be applied generally to members of the diverse family of enzymes. Currently, many PGT assays exploit the difference in solubility between a starting UDP-sugar substrate and the polyprenol-diphosphosugar product by analyzing reaction progress using liquid-liquid extractions^13^, solid-liquid extractions^14^ or chromatographic methods^15^. In these methods, enzymatic turnover is quantified using an isotop label ([^3^H] or [^14^C]) in the carbohydrate moiety of the UDP-sugar donor. For some PGTs, high-throughput assays using fluorescence have also been developed, however, these approaches require modification of the glycosyl phosphate donor, which is only feasible with PGTs such as MraY, where the fluorophore can be incorporated distal to the reacting groups^16^. Alternatively, multienzyme-based analyses, which couple UMP release to NAD^+^ generation, have been employed^17^.

In 2014, Promega launched the UDP-Glo assay that measures the activity of glycosyltransferase (GT) enzymes through the detection of UDP release, which is coupled to a bioluminescence signal^18^,^19^. The details of the UDP-Glo™ glycosyltransferase assay can be found in the technical manual from Promega Corporation. This assay has proved to be an excellent substitute for many of the labor-intensive assays commonly used for measuring the GT activities. The UDP-Glo assay is robust and highly sensitive and involves a one-reagent, one-step discontinuous assay ideally suited for measurement of the activities of GTs in a high-throughput fashion^18^,^19^. In the assay, UDP is converted to a nucleotide triphosphate (NTP), which is further converted to a stable bioluminescence signal by coupling to the luciferase/luciferin reaction components also present in the assay^18^,^19^. The assay has recently been used to study the activity^20^ and kinetic parameters^21^ of OGT (O-GlcNAc transferase). Additionally, the sugar specificity of the glucosyltransferase, GtfC, from *Streptococcus agalactiae* strain COH1 has been determined using the UDP-Glo assay^22^. Recently, a parallel strategy has been applied for the development of a rapid, luminescence-based homogeneous assay for the challenging class of PGT enzymes. In this case, the assay has been designated as UMP-Glo. In this assay the UMP by-product is converted to a stable luminescence signal (Fig. 1) by the action of a coupled enzyme system, again exploiting production of an NTP, which is coupled to a luciferase/luciferin reaction. As this assay detects the UMP by-product rather than the carbohydrate moiety, it should be versatile and readily applicable to PGT enzymes that use different sugar substrates. This approach considerably expands opportunities for studying PGTs, and circumvents the need for the synthesis of fluorescent or radiolabeled analogs of the sugar nucleotide substrates. Additionally, the assay allows for screening of enzymes for which the sugar substrate is unknown, by evaluating a range of potential sugar nucleotide substrates.

In this study, we present validation of the UMP-Glo assay by performing activity assays with PglC, a phosphoglycosyltransferase responsible for the initiation of the N-linked protein glycosylation pathway of *Campylobacter jejuni*^13^. PglC is an integral membrane protein with one predicted transmembrane helix (TMH)^11^. The enzyme transfers phospho-di-*N*-acetylbacillosamine (P-diNAcBac) from UDP-diNAcBac to an undecaprenol phosphate (Und-P) acceptor to generate Und-PP-diNAcBac, and releases UMP as the by-product (Fig. 1). The activity of PglC has previously been assessed using a radioactivity-based liquid-liquid extraction assay^13^. In the current study, we have employed the UMP-Glo assay to study PglC activity and compared the results with those from the radioactivity-based assay. The compatibility of the UMP-Glo assay with several important components of typical PGT reactions, including Triton X-100, DDM and DMSO, has been examined and a full kinetic analysis carried out. Studies with the UMP-Glo assay were also extended to a PGT with a similar predicted architecture (a single TMH and a cytosolic globular domain) but different substrate preference, PglC from *H. pullorum.* Additionally, the assay was used to examine WecA from *T. maritima,* a PGT with 11 TMHs, a dramatically different architecture from the PglC enzymes from *C. jejuni* and *H. pullorum*.

## Results

### Luminescence as a linear function of UMP concentration

**Figure 2:**
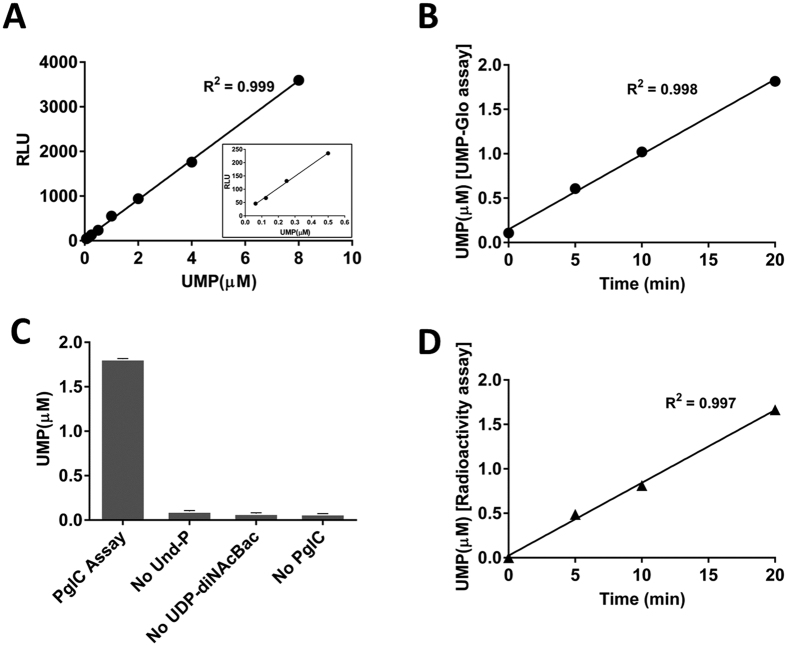
(**A**) Luminescence (RLU) as a linear function of UMP concentration. A standard curve demonstrated a linear correlation of RLUs with the concentration of UMP over the range of 62.5 nM to 8 μM. *Inset* is the correlation of RLUs with UMP concentrations over the range of 62.5 nM to 0.5 μM. (**B**) Time course of the PglC reaction using the UMP-Glo assay. Measurement of activity of PglC using the UMP-Glo assay showed that PglC activity was linear up to 20 min as measured. (**C**) PglC reaction and control experiments using UMP-Glo assay. Whereas PglC assays produced ~1.8 μM of UMP in 20 min, the control experiments exhibited luminescence that correspond to only 0.06 – 0.1 μM UMP. All the assays were carried out in duplicate. Error bars represent mean ± standard deviation (SD). (**D**) Time course of the PglC reaction using the radioactivity-based assay. Measurement of the activity of PglC using the radioactivity-based assay showed that activity was linear up to 20 min as measured. The rate of this reaction was similar to the rate measured using the UMP-Glo assay.

Analysis of PGT reactions using the UMP-Glo assay requires a standard curve relating the relative light units (RLU) to the concentration of UMP formed during the reaction. To this end, increasing concentrations of UMP were incubated with the UMP-Glo assay reagent and the resulting luminescence was measured as described (see Materials and Methods). As illustrated in Fig. 2A, the relationship between UMP concentration and luminescence signal is linear at concentrations as low as 62.5 nM and as high as 8 μM. Establishing this linear range is critical for determining the reaction conditions under which to perform kinetic analyses.

### Time course of the *C. jejuni* PglC reaction

With the linearity of the luminescence response confirmed, time course experiments were performed to investigate the activity of PglC using the assay. The reactions were performed with 20 μM Und-P, 20 μM UDP-diNAcBac^23^ and 1 nM PglC. These studies show that UMP production increases linearly for at least 20 minutes under the reaction conditions (Fig. 2B). To ensure that the observed UMP production was due to enzymatic turnover, the PglC assay was further investigated by systematic elimination of the assay components. The results clearly demonstrate that UMP production is not observed unless both substrates and the enzyme are present (Fig. 2C). The results of the UMP-Glo time course experiment were also compared to those obtained using the traditional radioactivity-based assay (Fig. 2D), which has been previously used to assess PglC activity^13^. The rates obtained using both assays were comparable, supporting that the UMP-Glo assay can be used to reproducibly perform PglC kinetic assays.

### Effect of Triton X-100, DDM and DMSO

**Figure 3:**
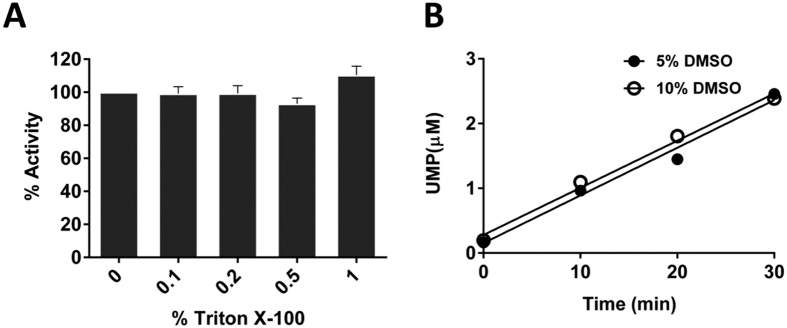
(**A**) Effect of Triton X-100 on the UMP-detection reagent. Standard solutions of Triton X-100 over the range of 0% to 1% in the presence of 2 μM UMP were used to measure luminescence. Up to 1%, Triton X-100 exhibited negligible effect on the UMP-detection reagent. Assays were performed in duplicate. Error bars represent mean ± standard deviation (SD). (**B**) Effect of DMSO on PglC-UMP-Glo assays. Effect of two different concentrations of DMSO (5% and 10%) on the rate of the PglC reaction was tested. The rate of both the reactions was linear and similar up to 30 min.

Next, the effects of additives such as DMSO and detergent were investigated to determine whether addition of these components influenced the assay readout. Inherent to the nature of both PGTs and the corresponding polyprenol-phosphate substrates, detergents are necessary for protein solubilization and in the enzymatic assay, however they are often incompatible with assay function and may result in background signal or suppression of signal. For PglC, the detergent Triton X-100 is used to solubilize the protein and polyprenol-linked substrate in the assay. Figure 3A shows that even concentrations as high as 1% Triton X-100 do not affect the luminescence signal obtained in the assay. The effect of another commonly used detergent for membrane protein solubilization, n-dodecyl β-D-maltoside (DDM), was also investigated on the assay readout. Concentrations of DDM as high as 1% in the assay do not influence the luminescence signal significantly (Figure S1). DMSO is another important reaction component, which is commonly used to solubilize the polyprenol-phosphate acceptor and small molecule inhibitors (*vide infra*). As can be seen in Fig. 3B, concentrations of DMSO as high as 10% do not significantly affect the luminescence signal of the assay.

### Kinetic parameters of PglC from *C. jejuni*

**Figure 4:**
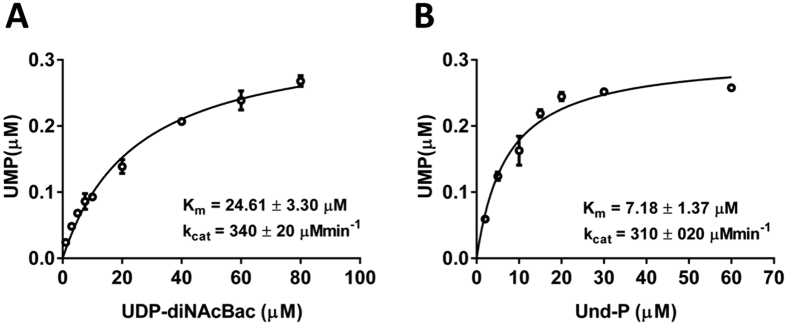
Kinetics of PglC using UMP-Glo assay. (**A**) PglC reactions were performed using various concentrations of UDP-diNAcBac (1–80 μM). Kinetic parameters were measured by fitting the data using the Michaelis-Menten equation. (**B**) PglC reactions were performed using various concentrations of Und-P (2–60 μM). Kinetic parameters were measured by fitting the data using the Michaelis-Menten equation. All the assays were carried out in duplicate. Error bars represent mean ± standard deviation (SD).

The highly sensitive and robust nature of the UMP-Glo assay allowed us to rapidly establish the kinetic parameters of PglC. Assays were performed at a fixed concentration of Und-P using variable concentrations of UDP-diNAcBac and vice versa in the presence of heterogolously-expressed PglC from *C. jejuni* (see Materials and Methods and Supporting Information Figure S2). The steady state kinetic parameters were measured by fitting the data using the Michaelis-Menten equation: K_m (UDP-diNAcBac)_ = 24.61 ± 3.30 μM; K_m (Und-P)_ = 7.18 ± 1.37 μM (Fig. 4). Similar k_cat_ values were measured, as expected, from both the experiments: 340 ± 20 min^−1^ and 310 ± 20 min^−1^ respectively.

### Time course of PglC from *H. pullorum*

**Figure 5:**
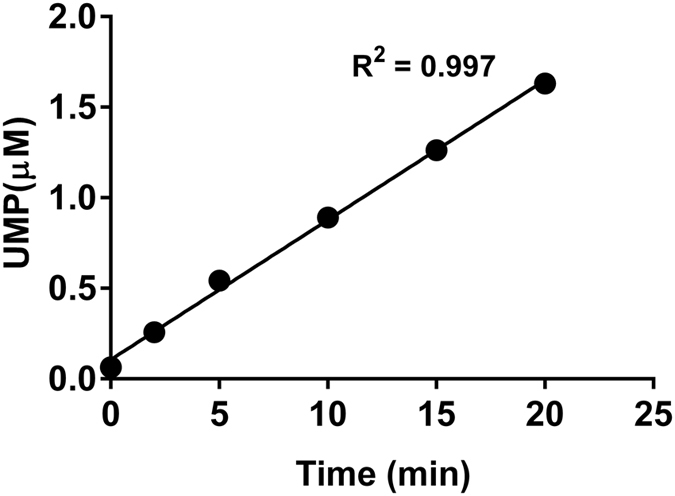
Time course of *H. pullorum* PglC reaction using UMP-Glo assay. Measurement of activity of PglC (*H. pu*) showed linear activity of the enzyme up to 20 min.

The UMP-Glo assay was also used to study the heterologously-expressed PglC from *H. pullorum* (*H. pu*) (Figure S3). This enzyme is predicted to have a similar membrane topology to the corresponding enzyme from *C. jejuni* and is suggested to transfer an unidentified HexNAc-phosphate from a UDP-HexNAc substrate, based on reported mass-spectrometry experiments^24^. In this study, we used the UMP-Glo assay to investigate whether the PglC (*H. pu*) acts on UDP-GlcNAc as a substrate. An initial time course assay using 0.3 μM PglC (*H. pu*), 20 μM UDP-GlcNAc and 20 μM Und-P demonstrates that the *H. pullorum* PglC catalyzes turnover of these substrates (Fig. 5), albeit at a slower rate compared to PglC from *C. jejuni*.

### Activity of WecA

**Figure 6:**
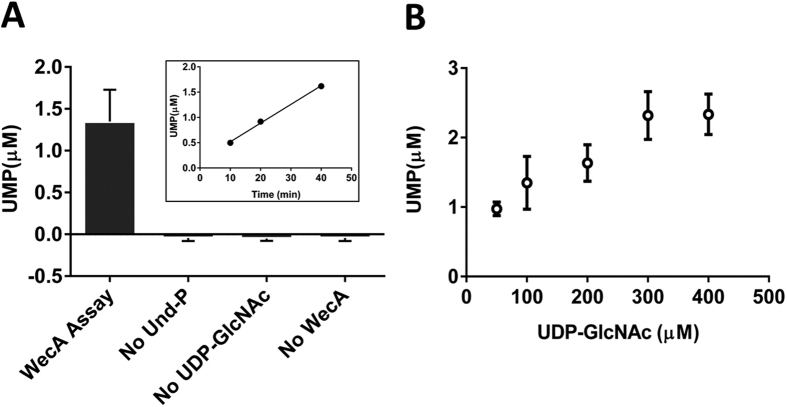
(**A**) WecA reaction and control experiments using UMP-Glo assay. Activity assessment of WecA using the partially purified enzyme showed ~1.3 μM of UMP production in 40 min. Under similar conditions, the control experiments exhibited negligible amount of UMP production. *Inset* shows the time course of WecA activity demonstrating a linear increase in UMP production as a function of time. (**B**) Effect of concentrations of UDP-GlcNAc on WecA activity. UMP production increased with increased concentration of UDP-GlcNAc up to 300 μM. All assays were carried out in duplicate. Error bars represent mean ± standard deviation (SD).

After validating the efficacy of the UMP-Glo reagent in measuring the activities of topologically similar PglCs from *C. jejuni* and *H. pullorum*, the assay was applied to assess the activity of WecA from *T. maritima*, a bacterial phosphoglycosyltransferase with a very different architecture. WecA includes 11 predicted transmembrane helical domains (TMHDs) and lacks a discrete soluble globular domain^4^,^25^. The enzyme transfers phospho-GlcNAc from UDP-GlcNAc to Und-P^4^,^26^,^27^, releasing UMP as a by-product. Bacterial members of this enzyme family play a crucial role in the biosynthesis of O-antigen, an essential component of lipopolysaccharide (LPS). Given the challenges associated with the purification of proteins containing multiple TMHDs, we initially employed the cell envelope fraction (CEF) of WecA in the pilot activity assays. However, application of the UMP-Glo reagent with the CEF resulted in significant background luminescence, even in the absence of the WecA substrates. The observed background luminescence signal was similar to the signal obtained in presence of both the substrates and CEF (See Supporting Information). These results suggest that the UMP-Glo assay reagent is incompatible with CEFs in measuring the activity of WecA. Attempts were made to purify the enzyme from the CEF utilizing the C-terminal His_6_ tag of WecA using Ni-NTA chromatography, and although the protein was found to bind poorly to the column, the resulting elution contained partially purified enzyme (Figure S4). This WecA preparation was then assayed in presence of 100 μM UDP-GlcNAc and 60 μM Und-P, and was found to be active (Fig. 6A) and in the time-course experiment, WecA exhibited linear increase in UMP production with increase in time (Fig. 6A insert). Control experiments performed by systematic elimination of Und-P, UDP-GlcNAc and the partially purified WecA from the assay did not produce significant amounts of UMP. The activity of WecA was also measured as a function of the concentration of UDP-GlcNAc. With increasing concentrations of the glycosyl phosphate donor substrate, UMP production increased (Fig. 6B), confirming that activity was substrate dependent. These results demonstrate that the UMP-Glo reagent can be used to assay the activity of WecA.

### Compatibility with inhibitor screening

**Figure 7:**
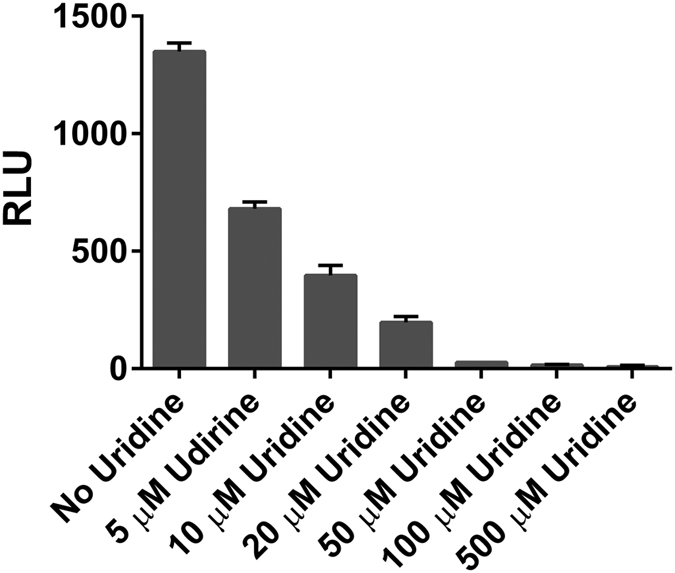
Effect of uridine on the UMP-Glo assay. Uridine was found to strongly inhibit the enzyme components present in the assay reagent. Experiments were performed in the presence of 3 μM UMP. The activity of the enzymes present in the UMP-Glo assay was greatly inhibited by the presence of uridine as low as 5 μM. In the presence of 50 μM uridine, the activity of the enzymes was inhibited by ~98%. All assays were carried out in duplicate. Error bars represent mean ± standard deviation (SD).

PGTs represent important targets for inhibitor design due to their centrality in bacterial glycan synthesis. Except for MraY, for which a high-throughput fluorescent assay has been developed^28^, advances in inhibitor development for the PGTs that transfer simple sugar phosphate moieties have been hampered by the traditional challenging and cumbersome kinetic assays discussed above. The 96-well plate-based format makes the UMP-Glo assay highly attractive for inhibitor screening. Recently, the UMP-Glo assay has proven its value in the evaluation of a set of uridine analogs for PglC inhibition^29^. However, whereas the UMP-Glo assay contributed to a facile assessment of inhibition of PglC by the larger molecular scaffolds, the smaller uridine fragments inhibited the UMP-Glo assay to some extent. In an attempt to quantify assay inhibition, increasing concentrations of uridine were added to a solution containing UMP, and processed using the UMP-Glo detection reagent (see Materials and Methods). Significant inhibition of the UMP-Glo assay alone was observed even at concentrations of uridine as low as 5 μM (Fig. 7). Complete inhibition of the UMP-Glo assay was observed at 50 μM or greater uridine concentrations, which are relevant concentrations in fragment screening. The issue of assay inhibition can be circumvented by performing the PGT inhibition assays in parallel with control experiments containing UMP-Glo reagents alone, to identify any potential inhibition of the UMP-Glo assay at specific concentrations of inhibitors.

## Discussion

This study demonstrates the potential of the UMP-Glo assay for PGT research. In a head-to-head comparison using PglC from *C. jejuni*, the UMP-Glo assay reported equally well on enzyme turnover as the radioactivity-based extraction assay, which has been a benchmark method to assay PGT reactions (Fig. 2D). A linear relationship between the UMP by-product and luminescence readout was observed under the standard reaction conditions used for PGT assays at concentrations ranging from 62.5 nM to 8 μM of UMP (Fig. 2A). The high sensitivity of the assay was demonstrated by the signal-to-background ratio, which was ~5 at UMP concentrations as low as 62.5 nM and ~350 at 8 μM UMP.

An important feature of the UMP-Glo assay is its compatibility with detergents such as Triton X-100 and DDM, which are essential additives for solubilizing PGTs and their lipophilic polyprenol-linked substrates. Moreover, this expands the potential scope of the UMP-Glo assay towards inhibitor screening, which often involves solubilization of inhibitors in DMSO. For this purpose, however, it is crucial to assess the impact of any added small molecules on the function of the UMP-Glo assay itself. It is demonstrated here that addition of decreasing amounts of uridine potently inhibits the UMP-Glo assay (Fig. 7), so the appropriate controls must be included when using the assay for inhibitor screening. The efficient throughput of the assay and compatibility with 96- and 384-well plate format is also advantageous for inhibitor screening, and allowed straightforward determination of the kinetic parameters for the *C. jejuni* PglC (Fig. 4).

The UMP-Glo assay has successfully been applied to measure the activity of three different PGTs (PglC from *C. jejuni* and *H. pullorum*, and WecA from *T. maritima*) that demonstrate different substrate specificity and/or protein topologies. This suggests that the assay will be extremely useful for investigating the thousands of different bacterial PGTs that have been identified using bioinformatics techniques^11^, but for which little is known about substrate specificity. As an example, we employed the UMP-Glo assay to investigate the activity of *H. pullorum* PglC enzyme, and determined that the enzyme was specific for turnover of UDP-GlcNAc under these conditions (Fig. 5). Additionally, given the challenges associated with the purification of membrane proteins, we investigated the level of WecA purity compatible with the UMP Glo assay. Cell envelope fractions (CEFs) are often used to study the activities of membrane proteins of interest, however we observed a significant luminescence background when the WecA CEF was combined with the UMP-Glo reagent, even in the absence of the substrates for WecA. However, partial purification of the WecA CEF revealed significant turnover in the UMP-Glo assay as evidenced by UMP production, which was observed only in presence of the partially purified enzyme and both substrates (Fig. 6A). These data imply that PGT enzymes require separation from native membrane-associated components in order to be studied using the UMP-Glo reagent. In summary, the ability of the UMP-Glo reagent to assess of the activity of both PglC from *C. jejuni* and *H. pullorum*, and WecA suggests that the scope of measuring activities for different PGT enzymes using the UMP-Glo assay is broad in nature.

## Conclusions

In summary, the efficacy of a newly developed luminescence-based assay for measuring the activities of PGT enzymes has been demonstrated in this work. The assay serves as an excellent alternative to the conventional radioactivity-based assays. Using PglC from *C. jejuni* as a model PGT enzyme, we have shown that the UMP-Glo assay recapitulates the radioactivity-based assay for measuring enzyme activity. However, in contrast to the radioactivity-based assay, the assay is simple, does not require preparation of specialized radiolabeled UDP-sugars (e.g. UDP-diNAcBac), is highly sensitive, rapid, and can be performed in a standard 96- or 384-well plate format. The high compatibility of the assay with commonly used additives in the PGT reactions such as Triton X-100, DDM and DMSO also reinforces the robustness of the assay particularly for membrane-bound proteins and for inhibitor screening. The validation of the UMP-Glo assay in measuring the activities of the PGT enzymes has also been corroborated using PglC from *H. pullorum* and WecA from *T. maritima*. While the assay is ideally suited for the evaluation of enzyme inhibitors, care has to be taken, for example with uridine-containing small molecules as they inhibit the UMP-Glo assay itself. In conclusion, the assay can be readily used in the identification of native UDP-sugar substrates and polyprenols for newly discovered as well as poorly understood PGT enzymes. Furthermore, the development of new PGT inhibitors should gain tremendous momentum with the availability of the UMP-Glo assay.

## Materials and Methods

### Materials

The prototype UMP-Glo assay kit was a generous gift from Promega. HEPES (99.9%) was obtained from Chem-Impex International. NaCl (>99%) and glycerol (>99%) were from Research Products International. Triton X-100 and DMSO (99.9%) were from Sigma-Aldrich, MgCl_2_.6H_2_O (100%) was from Mallinckrodt Chemicals. DDM (>99%) was purchased from Anatrace. 96-well half-area white plates were obtained from Corning. Ni-NTA resin was from Thermo Fisher. All other chemicals and bio-reagents were purchased at the purest grade available.

### Expression and purification of SUMO-PglC from *C. jejuni*

Heterologous expression of PglC equipped with an N-terminal His_6_ purification tag and a SUMO solubility tag was carried out in *E. coli* strain BL21-RIL. The protein was purified following the procedure as described previously^29^. Briefly, after cell lysis and removal of the cell debris, the cell envelope fraction (CEF, the membrane fraction) was prepared by high-speed centrifugation (150,000 × *g*). The CEF was solubilized overnight with 1% n-dodecyl β-D-maltoside (DDM). The detergent-solubilized PglC was then subjected to Ni-NTA affinity purification. The protein was eluted from the resin using 300 mM imidazole (See Supporting Information Figures S2 and S5). During all steps of purification, the DDM concentration was maintained at 0.03% (three times the CMC of the detergent).

### Expression and purification of PglC from *H. pullorum*

Heterologous expression of PglC from *H. pullorum* equipped with an N-terminal His_6_-SUMO purification and solubility tag was carried out in *E. coli* strain BL21-RIL. Purification of the protein was carried out in the same fashion as described for PglC from *C. jejuni* (See Supporting Information Figures S3 and S5).

### Expression and purification of WecA from *T. maritima*

Heterologous expression of WecA was carried out in BL21-RIL following the protocol as described previously^26^. Partial purification of the protein was carried out using Ni-NTA affinity chromatography utilizing the C-terminal His_6_-tag on the protein (See Supporting Information Figures S4 and S5). A detailed purification protocol of WecA is described in the supporting information.

### UMP-Glo assay

The prototype UMP-Glo assay kit was obtained from Dr. Hicham Zegzouti (Promega). The kit contained NTP (nucleotide triphosphate) detection substrate (lyophilized), nucleotide detection buffer, UMP-Glo solution and 10 mM UMP stock solution.

The UMP detection reagent was prepared following the manufacturer protocol. Both the nucleotide detection buffer and the NTP detection substrate were equilibrated at room temperature prior to use. The entire content of the nucleotide detection buffer was mixed gently and thoroughly with the lyophilized NTP detection substrate to generate the nucleotide detection reagent, which was aliquoted in small volumes and stored at −80 °C.

Before use in the assay, the nucleotide detection reagent was thawed on ice and equilibrated at room temperature. A 0.04 volume of the UMP-Glo solution was added to one volume of the nucleotide detection reagent to generate the UMP detection reagent. One volume of the UMP detection reagent was added to the equal volume of the reaction and mixed well to quench the reaction. Usually, the reactions were performed in a 15 μl reaction + 15 μl UMP detection reagent format. The quenched reaction mixture was immediately transferred to a 96-well plate (white, flat bottom, non-binding surface, half area, Corning Catalog No. 3992). To obtain consistent results, the generation of bubbles due to pipetting was avoided. The luminescence measurements were carried out using a SynergyH1 multi-mode plate reader (Biotek). The plate reader chamber was maintained at 25 °C. The 96-well plate was shaken inside the plate reader in the double orbital mode at 237 cpm for 16 min followed by incubation for 44 min, after which time the luminescence was measured. The gain of the luminometer and the integration time were kept at 137 and 0.5 sec respectively.

### Preparation of UMP standard curve

Standard UMP solutions were used to establish the reproducibility of the prototype UMP-Glo assay. UMP solutions ranging from 62.5 nM to 8 μM were made from 10 mM UMP stock solution (provided with the assay kit) using buffer containing 50 mM HEPES, 100 mM NaCl, pH 7.5, 5 mM MgCl_2_, 0.1% Triton X-100 and 10% DMSO. To 15 μl of a UMP standard solution, 15 μl of the UMP detection reagent was added and the corresponding luminescence was measured. A standard curve was plotted from the linear fitting (Y = 444.33X + 31.315, R^2^ = 0.999) of the data.

### PglC reactions using UMP-Glo assay

To measure the conversion in the PglC reaction, activity assays of PglC were carried out. Assays were performed in the presence of 1 nM of the enzyme and 20 μM of both the substrates, Und-P and UDP-diNAcBac. The Und-P stock was prepared in DMSO and the UDP-diNAcBac stock was prepared in H_2_O. The assay buffer contained 50 mM HEPES, 100 mM NaCl, pH 7.5, 5 mM MgCl_2_, 0.1% Triton X-100 and 10% DMSO (final). The assays were carried out at room temperature. PglC was pre-incubated in the assay buffer along with Und-P for 5 min. The reaction was initiated by the addition of UDP-diNAcBac. At various time points (0, 5, 10 and 20 min), 15 μl of the reaction were quenched with the 15 μl of the UMP-detection reagent and the luminescence was measured as described previously. The observed RLU values were converted to the concentrations of UMP using the standard UMP curve. The rate of the PglC reaction was obtained from linear fitting (Y = 0.0845X + 0.1483, R^2^ = 0.9973) of the data.

Since the UMP-Glo assay measures UMP release, it is critical to use ultrapure UDP-sugar substrates, devoid of any residual UMP, to minimize background luminescence. In addition, as the coupled assay is magnesium-dependent, metal ion chelators such as EDTA are incompatible with the UMP-Glo assay components.

The control experiments were also carried out in a similar fashion in the absence of one component of the assay at a time; namely, in the absence of Und-P, UDP-diNAcBac and PglC respectively. The assays were performed at room temperature for 20 min.

### PglC reactions using a radioactivity-based assay

Radioactivity-based assays were carried out in the presence of 1 nM of the enzyme and 20 μM of both the substrates, Und-P and UDP-[^3^H]- diNAcBac (5.4 mCi/mmol). The Und-P stock was prepared in DMSO and the UDP-[^3^H]-diNAcBac stock was prepared in H_2_O. The assay buffer contained 50 mM HEPES, 100 mM NaCl, pH 7.5, 5 mM MgCl_2_, 0.1% Triton X-100 and 10% DMSO (final). The assays were carried out at room temperature. PglC was pre-incubated in the assay buffer along with Und-P for 5 min. The reactions were initiated by the addition of UDP-[^3^H]-diNAcBac. At various time points (0, 5, 10 and 20 min), 20 μl of the reaction was quenched with 1 ml of 2:1 CHCl_3_:MeOH. The chloroform layer was washed with 3 × 400 μl of PSUP (Pure Solvent Upper Phase, composed of 15 mL CHCl_3_, 240 mL MeOH, 1.83 g KCl in 235 mL H_2_O). The resulting aqueous layers were combined with 5 mL EcoLite (MP Biomedicals) liquid scintillation cocktail. The organic layers were combined with 5 mL OptiFluor (PerkinElmer). Radioactivity of both the layers was measured by scintillation counting. Product formation was measured by scintillation counting of the organic phase which represented the UndPP-[^3^H]-diNAcBac. The rate of the PglC reaction was obtained from linear fitting (Y = 0.0819X + 0.0243, R^2^ = 0.997) of the data.

### Effect of Triton X-100 and DDM on the UMP-detection reagent

To measure the effect of Triton X-100 and DDM on the UMP detection reagent, several standard solutions were made that contained 2 μM UMP and varying concentrations of Triton X-100 and DDM ranging from 0% to 1%. After addition of the UMP-detection reagent to the solutions, luminescence was measured as described before. Luminescence from the sample that did not contain any detergent was expressed as 100% activity of the UMP detection reagent.

### Effect of DMSO on PglC-UMP-Glo assays

In order to study the effect of DMSO on the UMP detection reagent as well as on the PglC activity, time course assays of PglC were performed in presence of 5% and 10% DMSO. Reactions were performed at room temperature. At various time points (0, 10, 20 and 30 min), reactions were quenched with the equal volume of the UMP detection reagent and UMP generation was measured by the luminescence. The rate of both reactions was measured by linear fitting (Y = 0.0736X + 0.1539, R^2^ = 0.9833 and Y = 0.0728 + 0.2795, R^2^ = 0.990 respectively for 5% and 10% DMSO in the reaction) of the data.

### Kinetics of PglC using UMP-Glo assay

Kinetic parameters of PglC for both the substrates UDP-diNAcBac and Und-P were measured by performing assays at various concentrations of UDP-diNAcBac and Und-P and measuring UMP formation by the UMP-Glo reagent. Assays were performed in PglC assay buffer containing 50 mM HEPES, 100 mM NaCl, pH 7.5, 5 mM MgCl_2_, 0.1% Triton X-100 and 10% DMSO (final). Reactions were carried out at room temperature for 10 min. For measuring K_m_ of UDP-diNAcBac, the assays were performed using 1 nM PglC, 60 μM UndP and varying concentrations of UDP-diNAcBac (1–80 μM). For measuring K_m_ of Und-P, the assays were performed using 1 nM PglC, 80 μM UDP-diNAcBac and varying concentrations of Und-P (1–60 μM). The reactions were quenched with the equal volume of the UMP detection reagent and UMP generation was measured by the luminescence. The kinetic parameters were measured by fitting the data using Michaelis-Menten equation.

### Assays of PglC from *H. pullorum*

The time course of the *H. pullorum* PglC reaction was performed using 0.3 μM of the purified enzyme and 20 μM of both substrates, Und-P and UDP-GlcNAc in buffer containing 50 mM HEPES, 100 mM NaCl, pH 7.5, 5 mM MgCl_2_, 0.1% Triton X-100 and 10% DMSO (final). At various time points (0, 2, 5, 10, 15, 20 min), UMP-detection reagent was added to the assay and the generation of UMP was measured from the observed luminescence using the standard UMP curve. The rate of the reaction was measured by linear fitting (Y = 0.0771X + 0.106, R^2^ = 0.997) of the data.

### WecA activity assays

Assays were performed using partially purified enzyme in presence of 100 μM UDP-GlcNAc and 60 μM Und-P. Control assays were also carried out in the absence of Und-P, UDP-GlcNAc and WecA respectively. Assays were performed for 40 min at 65 °C in buffer containing 100 mM Tris-HCl, pH 8, 10 mM MgCl_2_ and 92.7 mM Triton X-100 and 10% DMSO (final)^26^. The reactions were quenched with the equal volume of the UMP detection reagent and UMP generation was measured by the luminescence. While studying the time course of the WecA assay, at various time points (10, 20 and 40 min), UMP-Glo reagent was added to the assay and luminescence was measured. While investigating the effect of concentrations of UDP-GlcNAc in WecA assay, reactions were performed in presence of 50, 200, 300 and 400 μM UDP-GlcNAc respectively.

### Uridine Inhibition assays

Standard uridine solutions of various concentrations (5, 10, 20, 50, 100 and 500 μM) were prepared in the assay buffer containing 50 mM HEPES, 100 mM NaCl, pH 7.5, 5 mM MgCl_2_, 0.1% Triton X-100, 10% DMSO (final) and 3 μM UMP. The reaction mixtures were treated with equal volume of the UMP-detection reagent and luminescence was measured.

## Additional Information

**How to cite this article**: Das, D. *et al*. A Rapid and Efficient Luminescence-based Method for Assaying Phosphoglycosyltransferase Enzymes. *Sci. Rep.* **6**, 33412; doi: 10.1038/srep33412 (2016).

## Supplementary Material

Supplementary Information

## Figures and Tables

**Figure 1:**
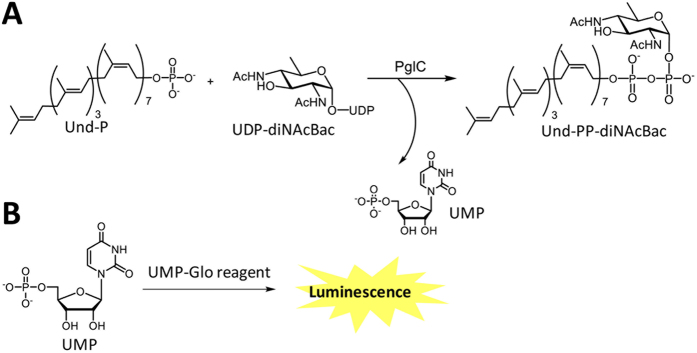
Phosphoglycosyltransferase reaction and UMP detection. (**A**) Reaction catalyzed by PglC to produce Und-PP-diNAcBac and release UMP as a by-product. (**B**) Detection of the UMP by-product by the UMP-Glo assay. UMP is converted to a luminescence signal by the UMP-Glo reagent.
