# Supplementary material for: A Rapid and Efficient Luminescence-based Method for Assaying Phosphoglycosyltransferase Enzymes
Source: Sci Rep. 2016 Sep 14;6:33412. doi: 10.1038/srep33412 (PMC5022061; doi:10.1038/srep33412)
Supplement: Supplementary Information [file srep33412-s1.pdf]

Supporting Information

A Rapid and Efficient Luminescence-based Method for Assaying Phosphoglycosyltransferase Enzymes

Debasis Das, Marthe. T. C. Walvoort, Vinita Lukose and Barbara Imperiali*

Department of Biology and Department of Chemistry

Massachusetts Institute of Technology

Cambridge, MA 02139, USA

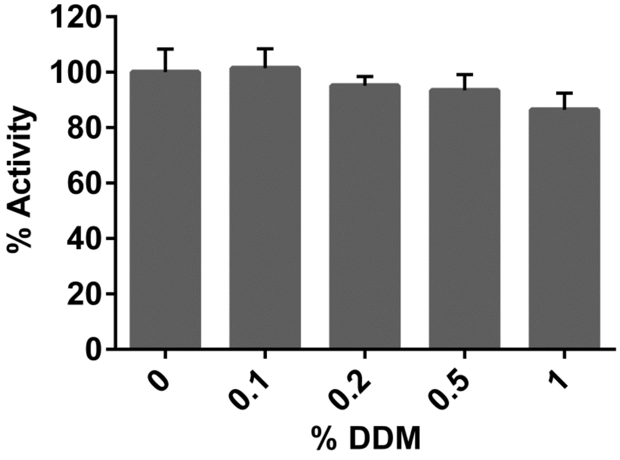

Figure S1. Effect of n-dodecyl β-d-maltoside (DDM) on the UMP-detection reagent. Standard solutions of DDM over the range of 0% to 1% in the presence of 2 μM UMP were used to measure luminescence. Up to 1%, DDM exhibited an almost negligible effect on the UMP-detection reagent. Assays were performed in duplicate. Error bars represent mean ± standard deviation (SD).

Purification of PglC from *C.jejuni*

Purification of PglC from *C.jejuni* was carried following protocol as described previously[1].

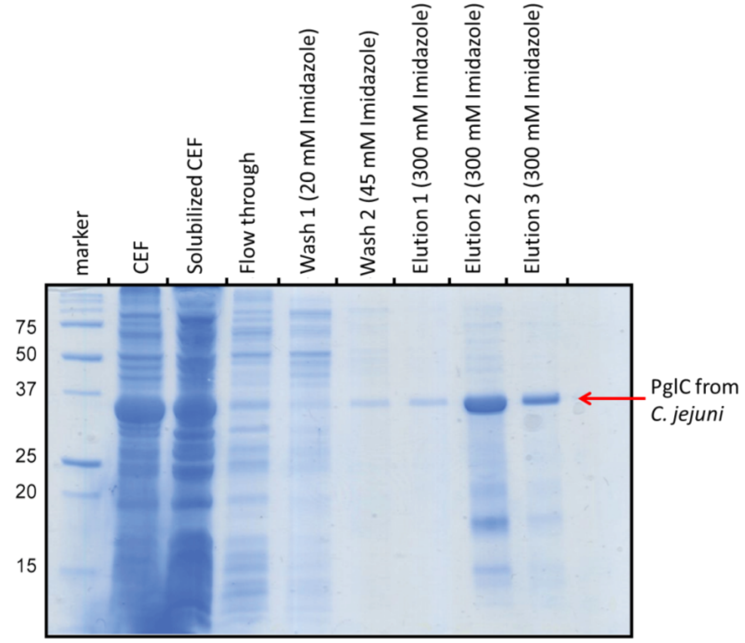

Figure S2. SDS-PAGE analysis of purification of PglC from *C. jejuni.* Presence of the protein in CEF was verified by SDS-PAGE. The protein was further purified by Ni-NTA affinity chromatography.

Purification of PglC from *H. pullorum.*

Purification of PglC from *H. pullorum* was carried out in the same fashion as PglC from *C. jejuni* following protocol as described previously[1].

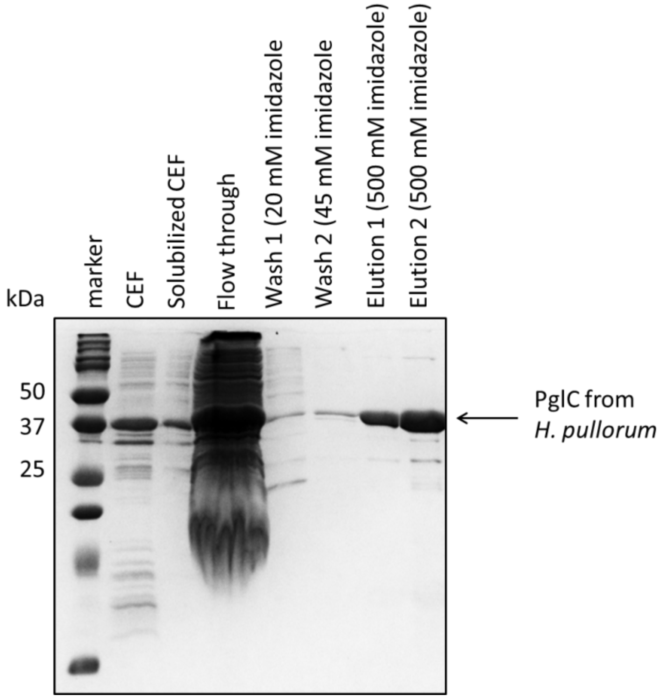

Figure S3. SDS-PAGE analysis of purification of PglC from *H. pullorum.* Presence of the protein in CEF was verified by SDS-PAGE. The protein was further purified by Ni-NTA affinity chromatography.

His_6-SUMO-PglC from *H. pullorum:*

HHHHHHGSLQDSEVNQEAKPEVKPEVKPETHINLKVSDGSSEIFFKIKKTTPLRRLMEAFAKRQGKEMDSL
RFLYDGIRIQADQAPEDLDMEDNDIIEAHREQIGGMYKNLIKPILDFILAFLLIIIFSPIILIVALLIKLKLGSPILFTQ
ERPGLNGKIFRIYKFRTMSDERDSKGDLLSDELRLKGFGKLIRKSSLDELPQLFNVLKGEMSFVGPRPLLVEY
LKLYNQEQAKRHNVKPGITGWAQVNGRNAISWEEKFKLDVYYVEHISFMLDCKILYMTFFKVLKRKDINSNT
NITMEKFTGNKSE

Expression and purification of WecA. WecA from *Thermotoga maritima* was used for this study. BL21-RIL cells were transformed with *wecA* in pET22b(+). The gene was modified to include expression of an N-terminal GB1 solubility-tag and a C-terminal His_6-tag. Heterologous expression and purification of WecA was carried out following the protocol as described previously[2]. A single colony from agar plate (kanamycin/carbenicillin) was grown overnight in 5

ml LB media with the same antibiotics at 37 °C. The overnight culture was transferred to 1L of LB media with the same antibiotics and grown at the same temperature to an OD of 0.7 at 600 nm. The culture was supplemented with 1 mM IPTG at 25 °C to induce protein expression. Cells were allowed to grow overnight at 25 °C and harvested at 3700 rpm for 30 min. Cells were further washed with 25 mM Tris-HCl, pH 7.5 containing 150 mM NaCl.

A 7 g batch of cells (from 1 L culture) was used for further manipulations. The entire process was carried out at 4 °C. The cells were lysed by sonication (2 X 90 sec, 1 sec ON - 2 sec OFF, 50% amplitude) in 50 ml buffer containing 25 mM Tris-HCl, pH 7.5, 150 mM NaCl, 10% glycerol, 2 mM 2-mercaptoethanol, 50 µl protease inhibitor, 25 µl DNAse and 25 mg lysozyme. The lysate was centrifuged at 9000 g for 45 min. The supernatant was further centrifuged at 140000 g for 1 h to obtain cell envelope fraction (CEF). The CEF was resuspended with 5 ml buffer containing 25 mM Tris-HCl, pH 7.5, 150 mM NaCl, 10% glycerol and 2 mM 2-mercaptoethanol. 2.5 ml of the resuspended CEF was further incubated with 6 ml of the same buffer supplemented with 2% DDM and 6 µl protease inhibitor cocktail (EMD Cat. #539134) for 2 h at 4 °C. The resulting solution was centrifuged at 150000g for 1 h. The supernatant was collected and diluted with 32 ml of buffer containing 25 mM Tris-HCl, pH 7.5, 150 mM NaCl, 10% glycerol, 2 mM 2-mercaptoethanol supplemented with 32 µl protease inhibitor cocktail to reduce the DDM concentration to 0.4%. The solution was incubated for 2 h with 1 ml of Ni-NTA (pre-equilibrated with 25 mM HEPES, pH 7.2, 150 mM NaCl, 10% glycerol, 0.2% DDM and 2 mM 2-mercaptoethanol, 10 mM imidazole). The flow through was separated, the column was washed with 20 column volume (c.v.) of the same column equilibration buffer followed by further wash with 20 c.v of the same buffer containing 20 mM imidazole. The bound proteins were eluted with the same buffer containing 300 mM imidazole and immediately desalted with desalting column (GE Healthcare). All the fractions were studied by SDS-PAGE. The samples obtained

after desalting was not pure however, the presence of WecA was confirmed as indicated by SDS-PAGE analysis by the arrow.

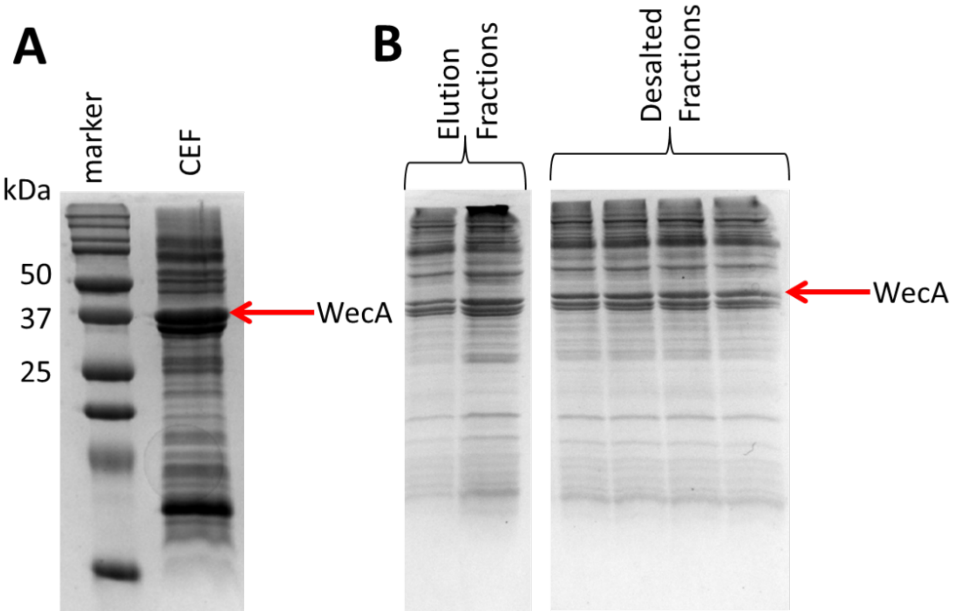

Figure S4. SDS-PAGE analysis of purification of WecA. (A) Presence of WecA in CEF was verified by SDS-PAGE. (B) Elution fractions and desalted fractions were impure however, contained WecA as indicated by arrow.

Sequence of GB1-WecA-His_6 from *Thermotoga maritima*:

MQYKLALNGKTLKGETTTEAVDAATAEKVFKQYANDNGVDGEWTYDDATKTFTVTEGSMWEAIISFFLTSVLSVFAKK
TEFLDRPDSRKSHGRAVPPVGGVSIFLTLLIFERDNPFFLFSIPLFLLGLLDDLFDLSYRIKLAVTALVAVWFSTAVTIEVSIF
GARIHPVFFVIWFVGMVNAFNVVDGLDGLLSGISLFSSLMIGERSLAFSIIGFLPWNLPDAKVFLGNSGSFLLGAYLSTAS
VVFFEGDLGYATLFLGFPFYEIVFSFVRRLVVKKNPFSPDEKHTHHVFSRKIGKWKTLLILVSFSLMFNLLGLSQKFYFIFLY
VVLCCVLLFTYCVLQR GNGNLKLLEHHHHHH

While investigating the activity of WecA using CEFs, a high background luminescence signal was observed that corresponds to ~4 µM UMP however, a similar signal was also observed in the control experiments lacking Und-P or UDP-GlcNAc. Therefore, the UMP-Glo reagent was not compatible for measurement of activity of WecA in unfractionated CEFs.

Western blot analysis of PglC from *C. jejuni*, PglC from *H. pullorum* and WecA from *T. maritima*. The proteins were first analyzed using 15% SDS-PAGE gels and then transferred to nitrocellulose membrane (Bio-Rad). Anti-His monoclonal antibody (Lifetein Cat. # LT0426) was used as the primary antibody and goat anti-mouse IgG, IgM (H+L) antibody conjugated with alkaline phosphatase (Thermo Fisher Cat. # 31328) was used as the secondary antibody. A One-step NBT/BCIP substrate solution (Thermo Fisher Cat. # 34042) was used for blot development.

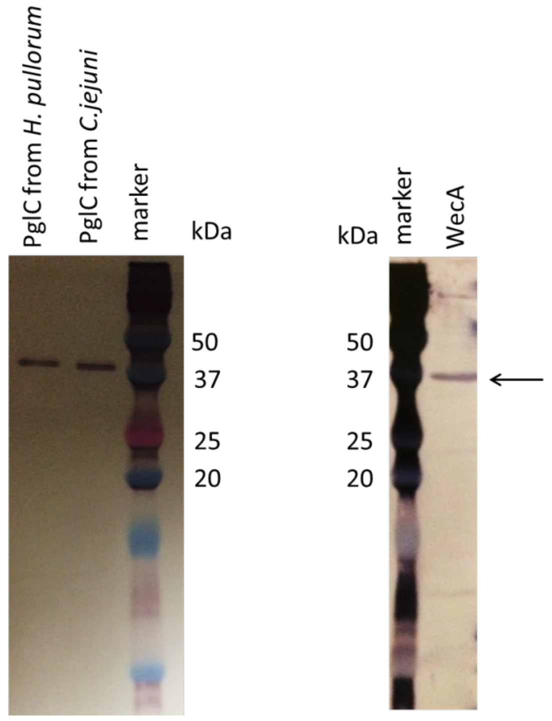

Figure S5. Western blot analysis of PglC from *H. pullorum,* PglC from *C. jejuni*, and *WecA* from *T. maritima*.

References

1 Walvoort, M. T. C., Lukose, V. & Imperiali, B. A modular approach to phosphoglycosyltransferase inhibitors inspired by nucleoside antibiotics. *Chem. Eur. J.* **22**, 3856-3864, (2016).

2 Al-Dabbagh, B., Mengin-Lecreulx, D. & Bouhss, A. Purification and characterization of the bacterial UDP-GlcNAc:undecaprenyl-phosphate GlcNAc-1-phosphate transferase WecA. *J. Bacteriol.* **190**, 7141-7146, (2008).
